# Report of the Surgeon General of the United States Army

**Published:** 1844-02-10

**Authors:** 


### REPORT OF THE SURGEON GENERAL OF THE UNITED STATES ARMY,

*For the year* 1843.

The Annual Report of the Surgeon General of the Army to the Department of War, shows a very satisfactory state of the Bureau under his charge. The fiscal operations have been conducted with a very judicious economy; the amount in hands for the year was $55,740 79; of which, on the 30th June, 1843, there remained an unexpended balance of $28,672 38.

The Report states: "During the last four or five years the average cost of the medical supplies has been about $2 60 per man, per year; for the last twelve months $2 22 per man. Below this amount we cannot go, without abridging the sick of some of the necessary comforts; but with this sum we have heretofore provided all the essential articles of medical stores, and we can continue to furnish every comfort and convenience which the officer or the enlisted soldier can reasonably expect or rightfully claim of the Government."

"The number of cases of indisposition which have been under treatment in the army, during the last twelve months, was 27,734; 26,820 of which occurred within the past year; 914 being cases that remained of the preceding year.

"Of the whole number of sick, 26,513 have been restored to duty, 309 have been discharged the service, 18 have deserted, and 160 have died; leaving, on the 30th of September, 726 still on the sick report.

"The mean strength of the army for the last twelve months has been about 9,863; and as the number of sick, during the same period, was 27,734, and the aggregate of deaths was 160, it will appear that the proportion of cases of indisposition to the number of men in service, was as $2\frac{81}{100}$ to 1, or 2.81 per cent.; the ratio of deaths to the number of men, as 1 to $61\frac{5}{8}$, or a fraction less than $1\frac{3}{8}$ per cent.; and the proportion of deaths to the number of cases treated, as 1 to $173\frac{7}{100}$, or $\frac{57}{100}$ per cent."

Besides the medical duties proper of the surgeons of the army, they have, and continue to be, zealously engaged in making meteorological observations at almost every point of our extended country. These observations are being collated by Mr. Espy, whose devotion to meteorological pursuits is so well known; and much information of a highly useful and practical nature may be obtained, as well to the philosophic physician, as to the agriculturist and commercial man. A more perfect knowledge of climatorial influences in the production or cure of disease, and in the perfection or degradation of the human race; a thorough investigation into the laws governing the winds; the causes of storms, and the indications of their approach; the *trajets* of hurricanes, &c., will, we make no doubt, be the rich fruits of well made observations in meteorology; in the making of which we are pleased to know, that the medical officers of our army take so active a part.

From a tabular statement accompanying the report, we find that of 27,734 cases, there were:

| | Cases. | Deaths. |
|---|---|---|
| **Fevers.** | | |
| Febris continua communis, | 69 | 1 |
| ——— intermittens quot., | 2,747 | 2 |
| ——— ——— tertian. | 2,445 | — |
| ——— ——— quartan. | 29 | 1 |
| ——— remittens, | 530 | 23 |
| ——— typhus, | 11 | 2 |
| ——— ——— icterodes, | 55 | 12 |
| ——— congestiva, | — | 4 |
| **Eruptive Fevers.** | | |
| Erysipelas, | 34 | — |
| Rubeola, | 5 | — |
| Scarlatina, | 1 | — |
| Varioloid, | 3 | — |
| **Diseases of the Organs connected with the Digestive System.** | | |
| Cholera, | 163 | — |
| Colica, | 371 | — |
| Cynanche parotidea, | 10 | — |
| Diarrhœa, | 2,291 | 9 |
| Dysenteria acuta, | 907 | 3 |
| ——— chronica, | 160 | 15 |
| Dyspepsia, | 100 | — |
| Enteritis, | 11 | — |
| Gastritis, | 54 | 1 |
| Hematemesis, | 3 | — |
| Hepatitis acuta, | 19 | — |
| ——— chronica, | 15 | 3 |
| Icterus, | 37 | — |
| Obstipatio, | 616 | — |
| Peritonitis, | 6 | 1 |
| Tonsillitis, | 221 | 1 |
| **Respiratory System.** | | |
| Asthma, | 13 | — |
| Bronchitis acuta, | 82 | — |
| ——— chronica, | 8 | 2 |
| Catarrhus, | 3,795 | 3 |
| Hæmoptysis, | 15 | — |
| Laryngitis, | 28 | 1 |
| Phthisis pulmonalis, | 31 | 26 |
| Pleuritis, | 237 | 4 |
| Pneumonia, | 132 | 11 |
| Influenza, | 1,348 | — |
| **The Brain and Nervous System.** | | |
| Apoplexia, | 11 | 4 |
| Cephalalgia, | 440 | — |
| Chorea, | 1 | — |
| Delirium tremens, | 156 | 11 |
| Epilepsia, | 82 | 1 |
| Mania, | 8 | — |
| Melancholia, | 1 | — |
| Meningitis, | 1 | 1 |
| Neuralgia, | 45 | — |
| Paralysis, | 14 | — |
| **The Urinary and Genital Organs.** | | |
| Calculus, | 1 | — |
| Cystitis, | 2 | — |
| Diabetes, | 8 | — |
| Enuresis, | 3 | — |
| Gonorrhœa, | 384 | — |
| Ischuria et Dysuria, | 19 | — |
| Nephritis, | 10 | 2 |
| Orchitis, | 73 | — |
| Strictura urethræ, | 17 | — |
| Syphilis primitiva, | 160 | — |
| ——— consecutiva, | 30 | — |
| Ulcus penis non syphilitica, | 16 | — |
| **The Serous Exhalant Vessels.** | | |
| Anasarca, | 11 | 1 |
| Ascites, | 1 | 1 |
| Hydrocele, | 1 | — |
| Hydrothorax, | 1 | 1 |
| **The Fibrous and Muscular Structures.** | | |
| Pernio, | 86 | — |
| Podagra, | 5 | — |
| Rheumatismus acutus, | 801 | — |
| ——— chronicus, | 314 | — |
| **Abscesses and Ulcers.** | | |
| Fistula, | 13 | — |
| Phlegmon et abscessus, | 802 | 1 |
| Ulcus, | 549 | — |
| Paronychia, | 32 | — |
| **Wounds and Injuries.** | | |
| Ambustio, | 97 | — |
| Amputatio, | 3 | — |
| Concussio cerebri, | 7 | 2 |
| Contusio, | 1,313 | 1 |
| Fractura, | 55 | — |
| Luxatio, | 52 | — |
| Punitio, | 12 | — |
| Sub-luxatio, | 350 | — |
| Vulnus incisum, | 603 | — |
| ——— laceratum, | 38 | — |

| | | |
|---|---|---|
| Vulnus punctum, | 88 | — |
| ——— sclopeticum, | 26 | 1 |
| **All other Diseases.** | | |
| Amaurosis, | 3 | — |
| Angina pectoris, | 1 | — |
| Cachexia, | 67 | — |
| Carcinoma, | — | 1 |
| Debilitas, | 91 | — |
| Ebrietas, | 385 | 2 |
| Hæmorrhois, | 182 | — |
| Hemeralopia et Nyctalopia, | 36 | — |
| Hernia, | 67 | — |
| Morbus cutis, | 66 | — |
| Morsus serpentis, | 3 | — |
| Necrosis, | 2 | — |
| Odontalgia, | 162 | — |
| Ophthalmia, | 600 | — |
| Ptosis, | 59 | — |
| Pericarditis, | 13 | 2 |
| Prolapsus ani, | 7 | — |
| Scorbutus, | 32 | 1 |
| Scrofula, | 8 | — |
| Splenitis, | 10 | — |
| Suicidium, | 1 | 1 |
| Toxicum, | 41 | — |
| Varix, | 10 | — |
| Vermes, | 4 | — |
| Epistaxis, | 2 | — |
| Old age, | — | 1 |
| Morbi varii, | 1,624 | — |
| | 26,820 | 160 |
| Remaining at last report, | 914 | |
| Total number treated, | 27,734 | |

[For this interesting abstract we are indebted to the kindness of Dr. McPhail, of the U. S. Army.—Ed.]

